# Seventy-five years of change: Long-term reorganisation of helminth communities in the rook (*Corvus frugilegus*) in Ukraine

**DOI:** 10.1016/j.ijppaw.2026.101289

**Published:** 2026-09-16

**Authors:** Valeriia Dupak, Valeriia Telizhenko, Oksana Greben

**Affiliations:** a Department of Fauna and Systematics of Vertebrates, I.I. Schmalhausen Institute of Zoology of the National Academy of Sciences of Ukraine, Kyiv, Ukraine; b Department of Evolutionary Morphology, I.I. Schmalhausen Institute of Zoology of the National Academy of Sciences of Ukraine, Kyiv, Ukraine; c Department of Parasitology, I.I. Schmalhausen Institute of Zoology of the National Academy of Sciences of Ukraine, Kyiv, Ukraine

**Keywords:** Parasites, Birds, Anthropogenic pressure, Temporal community reorganisation, Parasite community ecology, Ukraine

## Abstract

Population declines and extinctions affect all animal groups, yet long-term monitoring of helminths remains rare despite their ecological importance. This study analyses the helminth communities of the rook *Corvus frugilegus* Linnaeus, 1758 in Ukraine across three 25-year periods spanning over seventy-five years (1950–2024), using a total of 275 examined individuals, taking into account the effect of season on helminth communities. A total of 30 helminth species were identified (13 nematodes, 10 trematodes, 5 cestodes, and 2 acanthocephalans). Community composition differed significantly among historical periods (PERMANOVA, R^2^ ≈ 0.125, p < 0.001). Coverage-standardised Hill number diversity analyses revealed a pronounced depression of the helminth community in period 2 (1975–1999), probably linked with the peak of agrochemical intensification in Soviet agriculture. This period also characterised the complete absence of trematodes – a group particularly sensitive to agrochemical and other contaminations. Seasonal variation was also detected, mostly in the early period (1950–1974), but overall, the long-term pattern is much stronger than the seasonal one. Only four species persisted across all three periods, with host-specific and directly transmitted helminths showing the greatest stability. Interestingly, period 3 (2000-2024) is characterised by the emergence of nematodes *Eucoleus frugilegi* (Czaplinski, 1962) and *Microtetrameres helix* (Cram, 1927), which became dominant species. This likely reflects species range expansion or niche replacement following the collapse of earlier helminth communities. This study suggests a dynamic system rather than a simple decline and highlights the value of synanthropic species and institutional collections for reconstructing long-term dynamics of host–parasite systems under sustained anthropogenic pressure.

## Highlights

• Rook helminth communities were tracked across 75 years in Ukraine.
• Parasite diversity collapsed in 1975–1999, then recovered and reorganised by 2024.
• Four of 30 helminth species persisted across all three study periods.
• Two nematode species absent before 2000 became dominant in the most recent period.

## Graphical abstract

**Figure:**
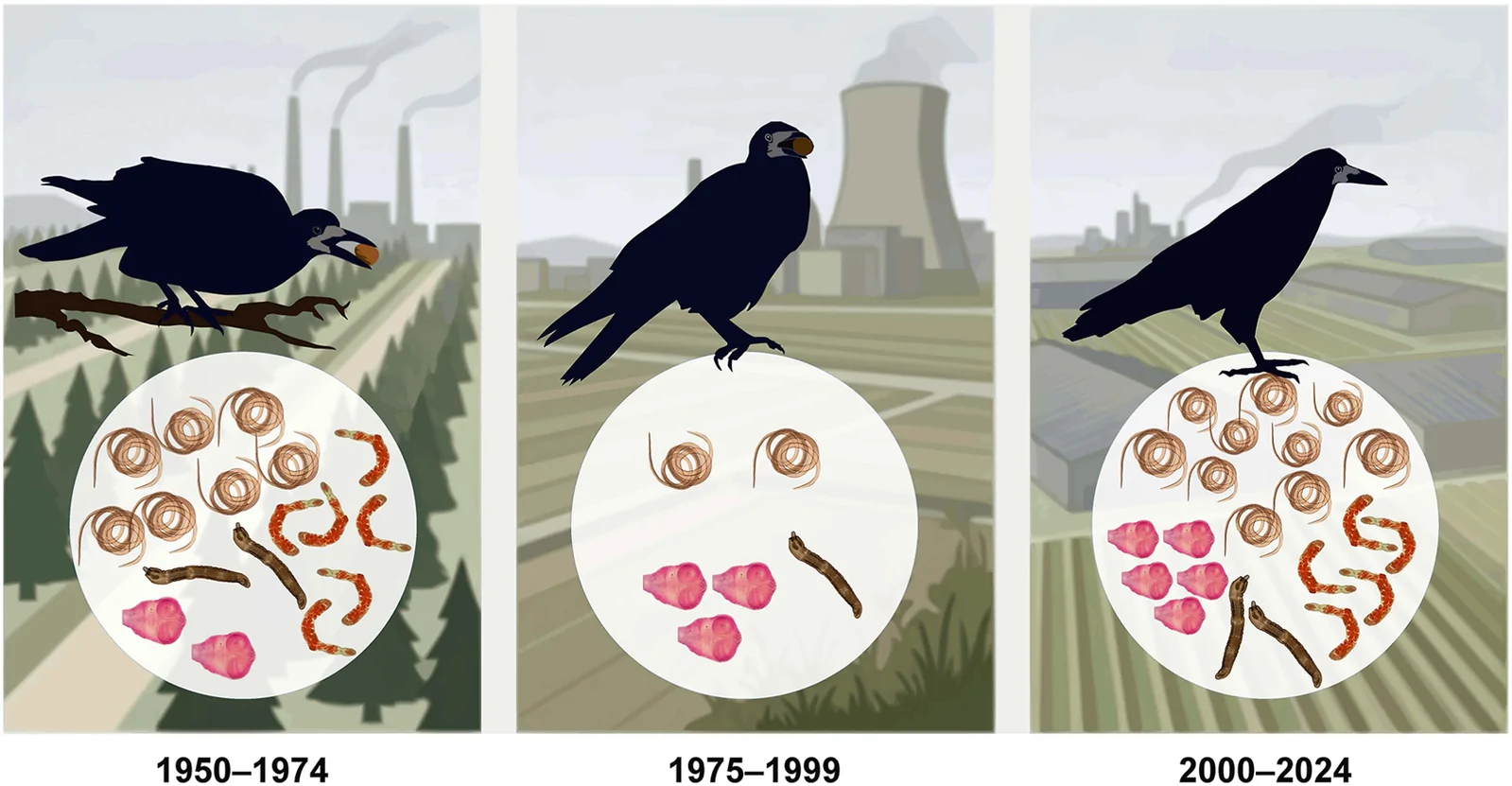

## 1 Introduction

Parasite communities, including helminths, are integral components of biodiversity and important elements of ecosystem functioning. Their structure and dynamics are closely linked to trophic networks, and parasites may account for up to 78% of trophic interactions in some food webs (Lafferty et al., 2006, 2008).

Environmental degradation, declines in the abundance of definitive and intermediate hosts, and the restructuring of trophic networks can strongly influence the composition of helminth communities and infection parameters of dominant species (Hechinger and Lafferty, 2005; Marcogliese et al., 2001; Sitko and Heneberg, 2020, 2021). However, parasites, unlike their hosts, are rarely studied from a long-term perspective and remain insufficiently integrated into discussions of the ongoing biodiversity crisis (Dougherty et al., 2016; Nichols and Gomez, 2011). This is not surprising, as there are no global long-term monitoring programmes for parasite communities, and the available body of literature addressing temporal changes in parasite communities is limited, methodologically heterogeneous, and often restricted to single host species, small geographic areas, or short time spans (Hammoud et al., 2025; Nichols and Gomez, 2011).

In the context of the ongoing mass extinction, the accumulation of data on temporal changes in parasite communities across different host groups (both vertebrates and invertebrates) is essential for developing a comprehensive understanding of ecosystem responses to global change. Among studies on the helminths of birds, there are very few publications explicitly focused on the temporal dynamics of helminth diversity and infection patterns (Hammoud et al., 2025; Sitko and Heneberg, 2020, 2021; Heneberg and Sitko, 2026). Moreover, some studies, although providing data on helminths occurrence over extended time periods, do not explicitly analyze long-term trends in helminth community change (Sitko and Heneberg, 2024; Syrota et al., 2021).

Although studies focusing specifically on bird helminths from a long-term perspective are scarce, the broader body of literature covering other host groups – invertebrates, mammals, fish, and others – makes it possible to outline several types of long-term changes in helminth fauna. Some studies report declines in prevalence, infection intensity, and overall helminth diversity (Behnke et al., 2021; Heneberg and Sitko, 2026). This may be linked to the simplification of trophic networks (Heneberg and Sitko, 2026). In other hosts, by contrast, an increase in the number of helminth species and range expansions of particular species have been documented (Franssen et al., 2014; Hayward et al., 2022). These upward trends are likely associated with climate change (Franssen et al., 2014; Hayward et al., 2022) and increasing host population density (Hayward et al., 2022). In some systems, parasite dynamics follow a non-linear pattern, with declines during certain decades followed by subsequent recovery that tracks the recovery of the host population itself (García-Gallego et al., 2023). Finally, in some hosts, helminth communities show temporal shifts in composition rather than a directional loss of diversity, with the disappearance of some species accompanied by the colonisation of others (Grzybek et al., 2015; Benovics et al., 2026).

Among passerine birds of agricultural and urban landscapes, the rook presents a particularly compelling model for investigating long-term changes in helminth communities. The species has a broad Palearctic distribution, with approximately 30% of its range falling within Europe (BirdLife International, 2025), and shows a strong association with agricultural land, forest-steppe zones, and riparian plains with scattered tree plantations, while readily nesting in towns and villages where sufficiently large trees are available (Benmazouz et al., 2021). The rook is among the most thoroughly studied birds in Ukraine, historically associated with large colonial nesting aggregations near cultivated fields, which were once considered to cause significant agricultural damage (Khranevych, 1925). Its broad diet, encompassing soil invertebrates, plant material, and increasingly anthropogenic food sources (Orłowski et al., 2009; Porter, 1979), creates multiple transmission pathways for helminths with the different life cycle strategies and intermediate host requirements, making it an ecologically rich host for helminth community studies.

The helminth fauna of the rook has been documented across parts of its European range since the mid-20th century, with records from Ukraine, Poland, and other parts of Eastern and Central Europe describing a taxonomically diverse fauna of nematodes, cestodes, trematodes, and acanthocephalans (Andreyko and Shumilo, 1970; Baruš et al., 1972; Bernard, 1960; Budkin, 1978; Chernobaj, 1969a; Iskova, 1975; Kornyushin et al., 1975; Pemberton, 1960; Rutkowska, 1973; Salamatin, 1999; Sitko and Heneberg, 2024; Spasskii and Oshmarin, 1939). However, the majority of existing studies represent geographically and temporally isolated surveys, and comparative analyses spanning multiple historical periods remain scarce. The capacity to track genuine temporal change in helminth community structure – rather than spatial variation among populations – requires a consistent geographic focus, standardised methodology, and access to archived material from earlier collection efforts, conditions that are rarely met simultaneously in the parasitological literature.

The present study addresses this gap by analysing helminth communities of the rook in Ukraine across three historical periods spanning over seven decades (1950–2024), using material from institutional collections to enable temporal comparisons at the regional level. We examine changes in species richness, prevalence, infracommunity structure, and community composition in the context of major ecological and land-use transformations in the region.

This approach offers insights into how helminth communities respond to long-term ecosystem change, highlighting synanthropic species as models for parasite adaptation to shifting environments, including anthropogenic pressure.

## 2 Materials and methods

### 2.1 Sampling of hosts and localities

We examined a total of 275 individuals of rooks. The data is based on both historical and modern material collected over 75 years (1950–2024). Most material (167 individuals) originated from the helminth collection of the I. I. Schmalhausen Institute of Zoology, National Academy of Sciences of Ukraine (Kyiv). Additionally, we examined 108 individuals during 2020–2024, collected as carcasses at winter roosts and near breeding colonies in summer, including contributions obtained through citizen science initiatives. A separate subset of rooks was collected in 2021 within the Askania-Nova Biosphere Reserve following a mass mortality event attributed to rodenticide poisoning in adjacent agricultural fields. The dataset used in the present study, comprising records of infected rooks and their helminths, together with quantitative infection parameters, collection dates and geographic coordinates, sampling regions, and the sex of helminths and hosts where known, is publicly available through the Global Biodiversity Information Facility (GBIF) (Greben and Dupak, 2025). In this dataset, the materialSampleID corresponds to the vial number under which the associated helminth material (nematodes, cestodes, trematodes, etc.) is stored in the helminth collection of the I. I. Schmalhausen Institute of Zoology.

**Fig. 1:**
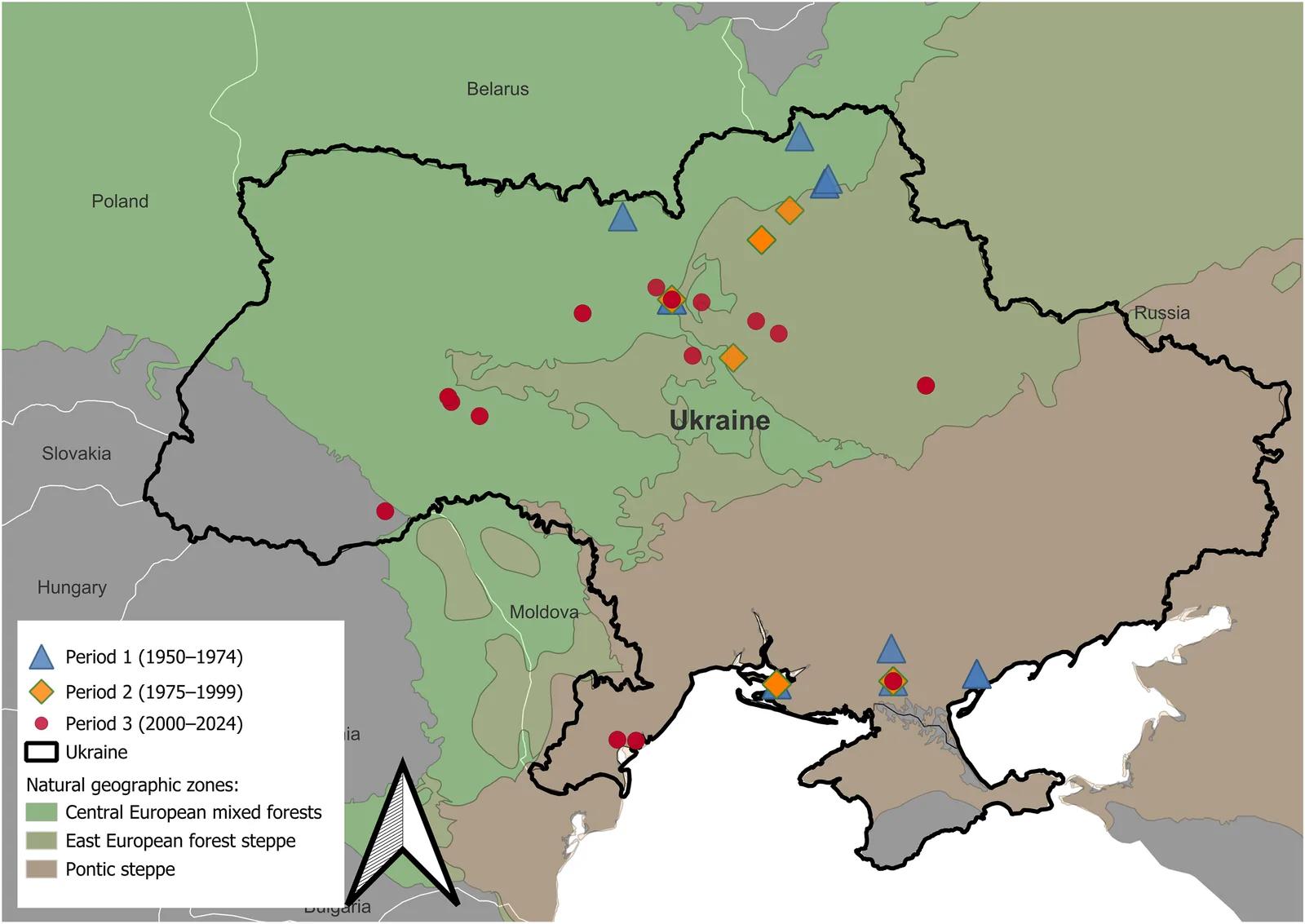
Sampling locations of hosts in Ukraine across three time periods (1950–1974, 1975–1999, and 2000–2024) in relation to major natural geographic zones.

The geography of the collection covered two main regions: northern and north-central Ukraine, spanning the Central European mixed forests and East European forest steppe zones, and the northern Black Sea coast, within the Pontic steppe zone (Fig. 1). Specimens from the Carpathian/Transcarpathian region were excluded, as bird populations there tend to be more geographically isolated due to the mountain barrier; the extreme eastern sampling points were also excluded, restricting the dataset to the lowland part of Ukraine and improving spatial homogeneity across periods. The largest host samples were obtained from Kyiv city (76 birds) and the northern Black Sea coast (Pontic steppe zone), mainly from the Black Sea Biosphere Reserve of NAS of Ukraine (78 birds) and Askania-Nova (48 birds). Kyiv and Askania-Nova contributed birds from all three periods.

### 2.2 Temporal coverage and seasonal sampling design

To assess historical change in infection patterns of helminth community and diversity of the rook, we partitioned all available records into three equal 25-year periods, aligned with key transitions in land use, agricultural policy and broader anthropogenic pressures in Ukraine. Although the ecological character of these pressures may have varied across sampling zones, key drivers, such as the agrochemical intensification and land-reclamation programmes of the 1960s, were implemented nationwide with comparable intensity, supporting a shared periodisation. Using equal time intervals helps to standardise sampling intensity across decades while anchoring comparisons to well-documented socio-environmental transitions that can affect helminth transmission pathways (intermediate-host availability, trophic structure, contamination loads, and host demography).

**Period 1 (1950–1974)**: Post-war reconstruction and rapid Soviet industrialisation/agricultural intensification, including intensive afforestation and plantation forestry in the mid-20th century, produced marked landscape transformation and escalating anthropogenic pressure (e.g., expansion of monocultures, drainage, and pesticide use) (Milakovsky et al., 2025). This sample included 98 individuals from 10 localities in 4 regions.

**Period 2 (1975–1999)**: Peak and aftermath of industrialisation followed by late-1980s structural transitions and the collapse of the Soviet agrarian system in the 1990s. This interval spans critical ecological inflexion points: rollback of certain agro-technologies, widespread land-use transitions, and partial farmland abandonment (Smaliychuk et al., 2016). During period 2, a total of 32 birds were sampled from 7 localities in 4 regions.

**Period 3 (2000–2024):** Post-socialist agricultural reorganisation and consolidation, with a transition from more heterogeneous, smallholder-oriented practices to agroholding-led intensification and “chemicalization” (increased use of fertilisers, pesticides, and rodenticides) (Moldavan and Pimenova, 2021; Smaliychuk et al., 2016). The period also encompasses superimposed regional climate trends of the 2000s–2020s, including a documented warming trend of approximately 0.64°C per decade across Ukraine (Boychenko and Maidanovych, 2024). This sample contains 145 rooks from 15 localities in 7 regions.

The three periods are hereafter referred to as P1, P2, and P3, respectively.

To assess the seasonal and trophic drivers of infection parameters by different helminth species, we additionally analysed differences in helminth community composition between wintering and summer populations of the rook. This approach is justified, first, by the migratory behaviour of the species. Rooks appear in large numbers in Ukrainian settlements from the second half of October, mainly in November; a substantial proportion of wintering individuals originates from eastern regions of Eurasia (Russia and Kazakhstan), whereas birds breeding in Ukraine may migrate to eastern and southern regions for wintering (Poluda and Tsukanova, 2012). Second, the rook is an omnivorous species whose diet varies markedly with seasonal resource availability (Orłowski et al., 2009; Porter, 1979). During the warm period, increased consumption of invertebrates is expected to enhance exposure to helminths via intermediate hosts. In contrast, during the cold season, birds predominantly shift to plant-based foods and anthropogenic food sources, which likely reduces the intake of invertebrates. Under such conditions, a partial decline in infection intensity may occur due to limited acquisition of intermediate hosts, resulting in a functional “partial deworming” effect.

Based on seasonal variation in diet and migratory status, all hosts were therefore assigned to two seasonal groups: a summer sample (April–October; n = 102 birds) and a winter sample (November–March; n = 173 birds).

### 2.3 Collection and identification of helminths

Dead birds were transported to the laboratory, where a full-body helminthological necropsy was performed. We examined the subcutaneous tissue, body cavity, oesophagus, stomach, intestines, cloaca, bursa of Fabricius, liver, gall bladder, spleen, lungs, trachea, bronchus, air sacs, kidneys, and oviducts using a stereomicroscope. Helminths were collected, rinsed in saline, and fixed in 70% ethanol. Specimens were examined under a light microscope (AmScope T690B) and identified based on morphological characteristics according to previous publications (Czaplinski, 1962; Iskova, 1979; Lisitsyna, 2019; Salamatin, 1999; Sharpilo and Iskova, 1989; Skrjabin, 1952a;Skrjabin, 1952b; Skrjabin et al., 1957; Skrjabin and Sobolev, 1963; Smogorzhevskaya, 1990; Sonin, 1968; Spasskaja, 1966; Spasskaja and Spassky, 1977).

Prior to examination, nematodes were washed in distilled water for 10–30 min and cleared in lactophenol for 10–30 min, depending on worm size. Acanthocephalans were washed in distilled water and cleared in Berlese medium. Trematodes and cestodes were stained with iron acetocarmine, dehydrated through a graded ethanol series, cleared in clove oil, and mounted in Canada balsam (Lutz et al., 2017). Some cestode specimens consisting only of proglottids or scolices were cleared in Berlese's medium to study the armament of the scolices and the copulatory apparatus. Collection material was prepared using the same clearing and staining procedures as applied to freshly collected specimens. The identification was carried out based on morphological features throughout all three periods. Material from P1 and P2 originated from historical collections (permanent slides, formalin- or low-quality ethanol-fixed specimens), precluding molecular analysis due to DNA degradation; morphological identification was therefore applied uniformly, including for P3, to ensure methodological consistency across periods.

### 2.4 Statistical methods

The dataset consisted of individual host records with helminth counts recorded separately for each helminth taxon. The distribution of examined hosts was summarized across historical periods, seasons, regions, sex, and age classes using frequency tables. Then we used cross-tabulations to describe the joint distribution of the historical period with season, region, host's sex, and age. These summaries were utilized to describe the distribution of examined hosts across key sampling categories.

To characterize infection patterns in the host population, we calculated metrics at two levels: host-level parameters describing infracommunity structure within individual birds, and parasite-level parameters that describe the distribution and abundance of each helminth taxon.

At the host level the following parameters were calculated: (i) proportion of infected hosts (with Wilson 95% confidence intervals); (ii) mean total helminth burden per host (with bootstrap confidence intervals); (iii) mean helminth richness per host (with bootstrap confidence intervals).

For each helminth species, the parasitological parameters were calculated following Bush et al. (1997): (i) prevalence, defined as the proportion of infected hosts among all examined hosts, with 95% confidence intervals calculated using Wilson's binomial method; (ii) mean intensity, corresponding to the average helminth count among infected hosts only; and (iii) mean abundance, calculated as the mean helminth count among all examined hosts, including uninfected individuals. Confidence intervals for mean intensity and mean abundance were estimated using non-parametric bootstrap resampling (percentile method, 2000 replicates).

Additionally, we calculated the dispersion index as the variance-to-mean ratio of helminth abundance per host, where values equal to 1 indicate random (Poisson) distribution and values greater than 1 reflect aggregated distribution of helminth among hosts.

Parasite-level metrics were calculated for the full dataset and separately by season. All statistical analyses were conducted in R (v. 4.x.x) using the tidyverse framework (Wickham et al., 2019).

Temporal variation in the diversity of the helminth community was evaluated using Hill numbers (q = 0, 1, 2). The analysis was performed using the iNEXT R package (Chao et al., 2014; Hsieh et al., 2016), with coverage-based standardization to account for unequal sampling effort among historical periods. Diversity was calculated from pooled helminth abundances per period, and bootstrap procedures were used to obtain 95% confidence intervals. To further explore potential seasonal influences, diversity was also estimated separately for each combination of historical period and season.

To analyze changes in helminth community composition across historical periods, Bray–Curtis dissimilarities were computed from abundance data (Bray and Curtis, 1957), and Sørensen dissimilarities were used for presence/absence comparisons (Sørensen, 1948). Non-metric multidimensional scaling (nMDS) was applied to the Bray–Curtis and Sørensen distance matrices to reduce the complexity of the multivariate relationships into two-dimensional space. The usage of both distance methods allowed us to capture intensity-based (Bray–Curtis) and compositional (Sørensen) dissimilarities. There were six outlier samples, which were removed for visualization (hosts with ID numbers 5CF, 31CF, 124CF, 159CF, 270CF). These samples occupied extreme positions in the nMDS ordination space and were excluded to improve clarity of the plot. Further analyses were performed on the full dataset.

To assess significant differences in community composition between historical periods and seasons, PERMANOVA was used on Bray-Curtis and Sørensen distances with permutation tests to assess group separation (Anderson, 2001). Because unequal sample sizes can influence PERMANOVA results in unbalanced designs (Anderson and Walsh, 2013), we performed additional analyses using repeated random subsampling to equalize group sizes among historical periods prior to PERMANOVA. Then, to ensure that significant differences detected by PERMANOVA were not confounded by differences in the variability of communities within groups, a multivariate dispersion test (betadisper) was applied as a check for homogeneity of dispersions (Anderson, 2006). Community differences among historical periods and seasons were additionally evaluated with analysis of similarities (ANOSIM) based on Sørensen dissimilarities (Clarke and Green, 1988).

To check whether temporal patterns could be confounded by heterogeneity in sampling localities among periods, we performed two additional analyses. First, we refitted the PERMANOVA with sampling region (oblast) as a covariate and tested the marginal effect of historical period after accounting for region. Second, we repeated the among-period comparison separately within the two localities sampled in more than one period (Kyiv and Askania-Nova), after restricting each site to the periods represented by at least eight hosts.

Next, we used indicator species analysis (IndVal) to identify helminth taxa that were significantly associated with specific historical periods (Dufrêne and Legendre, 1997). Similarity percentage analysis (SIMPER) was applied to the Sørensen (presence/absence) dissimilarities to quantify the contribution of individual helminth species to compositional differences between each pair of historical periods (Clarke, 1993).

We selected three focal species based on the following criteria: the highest prevalence combined with occurrence in each period. For these species (*Acuaria anthuris* (Rudolphi, 1819) and *Baruscapillaria resectum* (Dujardin, 1845), *Spiniglans affinis* (Krabbe, 1869)), prevalence was calculated separately for each historical period, with Wilson 95% confidence intervals. To evaluate temporal changes in infection probability, we fitted logistic regression models with binomial error distribution for each focal species. Infection status (infected vs. uninfected) was used as the response variable, and historical period as a categorical predictor. We also compared additive models that included historical period and season as independent effects with interaction models that additionally tested whether temporal patterns differed between seasons. Odds ratios with 95% confidence intervals were obtained from exponentiated model coefficients.

All scripts used for the analyses are available on GitHub: https://github.com/vlr4/rook-parasites-ukraine.

## 3 Results

### 3.1 Helminth species composition

A total of 2287 helminth specimens belonging to 30 species were found in all examined material: 13 nematode species, 5 cestode species, 10 trematode species, and 2 species of acanthocephalans. The species composition of the recorded helminths and their sites of parasitism are presented in Table 1. Among the nematodes, the identification of specimens of the genus *Microtetrameres* was challenging in cases where only females were found in the absence of males, or males of both species were present, but the females could be distinguished only by size and lacked sufficient morphological features for reliable species-level identification. In the case *Spirurida* gen. sp., the material consisted of larval stages.

**Table 1:** Recorded helminth species and their sites of infection.

Species	Site of infection
**Plathyhelmintes, Trematoda**	

*Apophallus muehlingi* (Jägerskiöld, 1899)	intestine
*Echinostoma revolutum* (Fröhlich, 1802)	intestine
*Laterotrema vexans* (Braun, 1901)	cloaca
*Lyperosomum alaudae* (Shtrom and Sondak, 1935)	intestine
*Lyperosomum longicauda* (Rudolphi, 1809)	liver
*Plagiorchis elegans* (Rudolphi, 1802)	intestine
*Prosthogonimus cuneatus* (Rudolphi, 1809)	bursa Fabricii
*Prosthogonimus ovatus* (Rudolphi, 1803)	bursa Fabricii
*Strigea sphaerula* (Rudolphi, 1803)	intestine
*Urogonimus turdi* (Yamaguti, 1939)	intestine

**Plathyhelmintes, Cestoda**	

*Dilepis undula* (Schrank, 1788)	intestine
*Passerilepis crenata* (Goeze, 1782)	intestine
*Passerilepis stylosa* (Rudolphi, 1809)	intestine
*Spiniglans affinis* (Krabbe, 1869)	intestine
*Spiniglans constricta* (Molin, 1858)	intestine

**Acanthocephala, Palaeacanthocephala**	

*Plagiorhynchus cylindraceus* (Goeze, 1782)	intestine
*Sphaerirostris picae* (Rudolphi, 1819)	intestine

**Nematoda, Enoplea**	

*Baruscapillaria resecta* (Dujardin, 1845)	intestine
*Eucoleus contortus* (Creplin, 1839)	oesophagus
*Eucoleus corvicola* Wassilkowa, 1930	oesophagus
*Eucoleus frugilegi* (Czaplinski, 1962)	oesophagus

**Nematoda, Chromadorea**	

*Acuaria anthuris* (Rudolphi, 1819)	gizzard, under koilin layer
*Cylicodontophorus bicoronatus* (Looss, 1900)	intestine
*Diplotriaena tricuspis* (Fedtschenko, 1874)	body cavity
*Dispharynx nasuta* (Rudolphi, 1819)	gizzard, under koilin layer
*Eufilaria delicata* Supperer, 1958	oesophagus
*Microtetrameres contorta* (Weidman, 1913)	proventriculus wall and mucosa
*Microtetrameres helix* Cram, 1927	proventriculus wall and mucosa
*Microtetrameres inermis* (Linstow, 1879)	proventriculus wall and mucosa
*Spiruridae* gen. sp. (L3)	intestine

During P1 (1950–1974), 439 helminth specimens belonging to 17 species were collected (7 nematodes, 2 cestodes, 6 trematodes, and 2 acanthocephalans). In P2 (1975–1999), 221 specimens of 6 species (2 nematodes, 3 cestodes, and 1 acanthocephalan) were obtained in birds. The helminth community recorded in the most recent period (2000–2024) comprised 1627 specimens from 23 species (11 nematodes, 5 cestodes, 5 trematodes, and 2 acanthocephalans).

Only four helminth species were recorded in all three periods: the nematodes *A. anthuris* and *B. resectum*, the cestode *S. affinis*, and the acanthocephalan *Sphaerirostris picae* (Rudolphi, 1819). Of these, only *A. anthuris*, *B. resectum*, and *S. affinis* belong to the core species, with prevalence exceeding 18%. Notably, among the helminths recorded in the most recent period, two nematode species (*Eucoleus frugilegi* (Czaplinski, 1962) and *Microtetrameres helix* (Cram, 1927) showed high intensity and prevalence of infection and are common parasites of the rook, yet they were not recorded at all in earlier years (1950–1999).

### 3.2 Host- and infracommunity-level infection parameters

Overall**,** helminths were recorded in 210 of 275 examined rooks (76.4%). From the sample of birds in P1, 68 out of 98 individuals were infected, corresponding to a prevalence of 69.4%. In P2, 59.4% of birds were infected. In the most recent period, the prevalence of infection was the highest, reaching 84.8%.

**Fig. 2:**
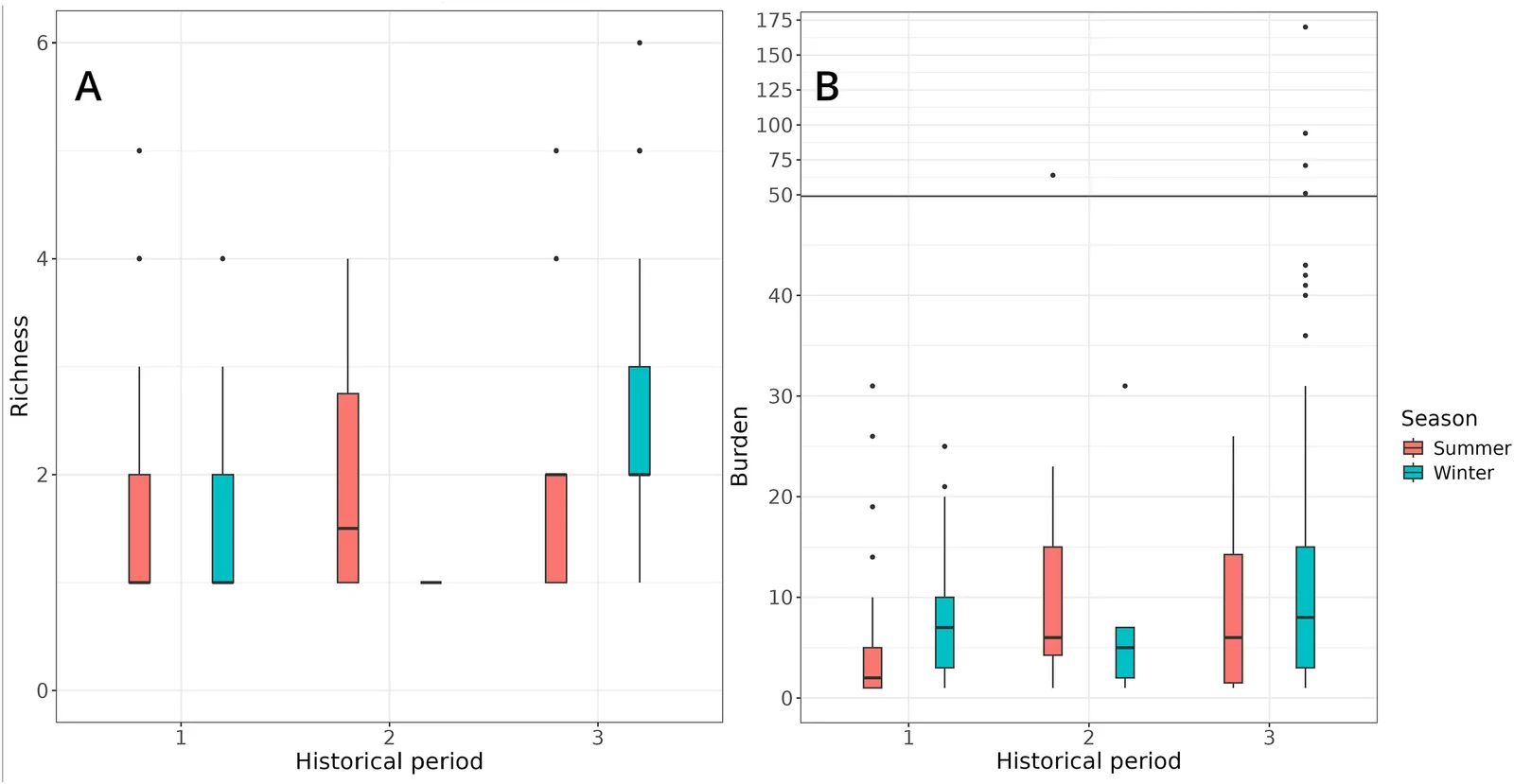
Host-level helminth metrics: (A) helminth species richness per host; (B) helminth burden per host.

Helminth species richness in contributed samples per host ranged from 1 to 6 species, with a mean of 2.07 species per host (SD = 1.19). Most of the infected hosts harboured one or two helminth taxa, whereas infections with three or more species were less common. Helminth species richness (Fig. 2A) in infected birds from P1 ranged from 1 to 5 (mean 1.54; median 1), and in P2 from 1 to 4 (mean 1.63; median 1). In P3, species richness in helminth infracommunities ranged from 1 to 6 (mean 2.42; median 2). Mean helminth burden was 10.89 individuals per host (SD = 16.67), indicating an overdispersed distribution (Fig. 2B).

**Fig. 3:**
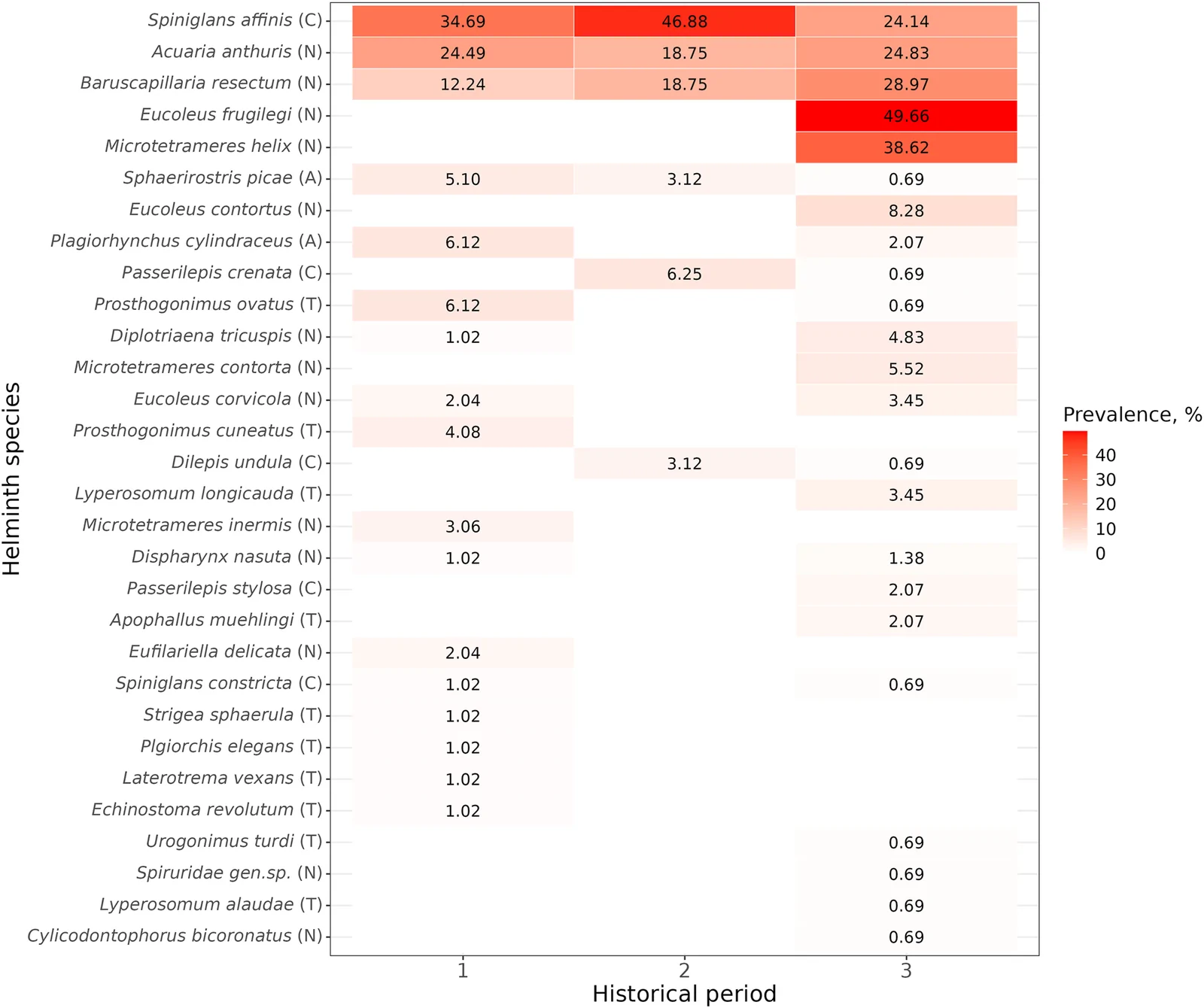
Temporal variation in prevalence among helminth taxa across the three study periods. Species are labelled with their taxonomic group: A – Acanthocephala; C – Cestoda; N – Nematoda; T – Trematoda.

Infection parameters across the entire bird sample varied among helminth taxa. Prevalence ranged from 0.4% to 30.5%, mean intensity from 1 to 7.4, and mean abundance from 0.004 to 1.96 per host. Prevalence patterns varied markedly among taxa and periods (Fig. 3). Among core species, *B. resectum* showed a consistent increase across periods (12.1–28.9%), while *S. affinis* peaked in P2 (46.9%) before declining sharply in P3 (24.1%); *A. anthuris* remained comparatively stable throughout. The most striking pattern was the emergence of *E. frugilegi* and *M. helix* – entirely absent in P1–P2 but reaching high prevalence in P3 (up to 49.7% for *E. frugilegi* and 38.6% for *M. helix*) – alongside a broader increase in rare, period-specific taxa recorded only in P3.

Helminth abundance was typically aggregated among hosts, as indicated by dispersion indices (variance-to-mean ratios) exceeding 1 for most helminth taxa (range 0.99–43.11). This also supports a previously assessed pattern where a small number of hosts harbour disproportionately high parasite burdens. Detailed numerical summaries are provided in Supplementary Table S1 (richness and burden summarized by period and season), Supplementary Table S2 (dispersion indices) and Supplementary Table S3 (abundance, prevalence, intensity).

### 3.3 Temporal changes in helminth diversity

**Fig. 4:**
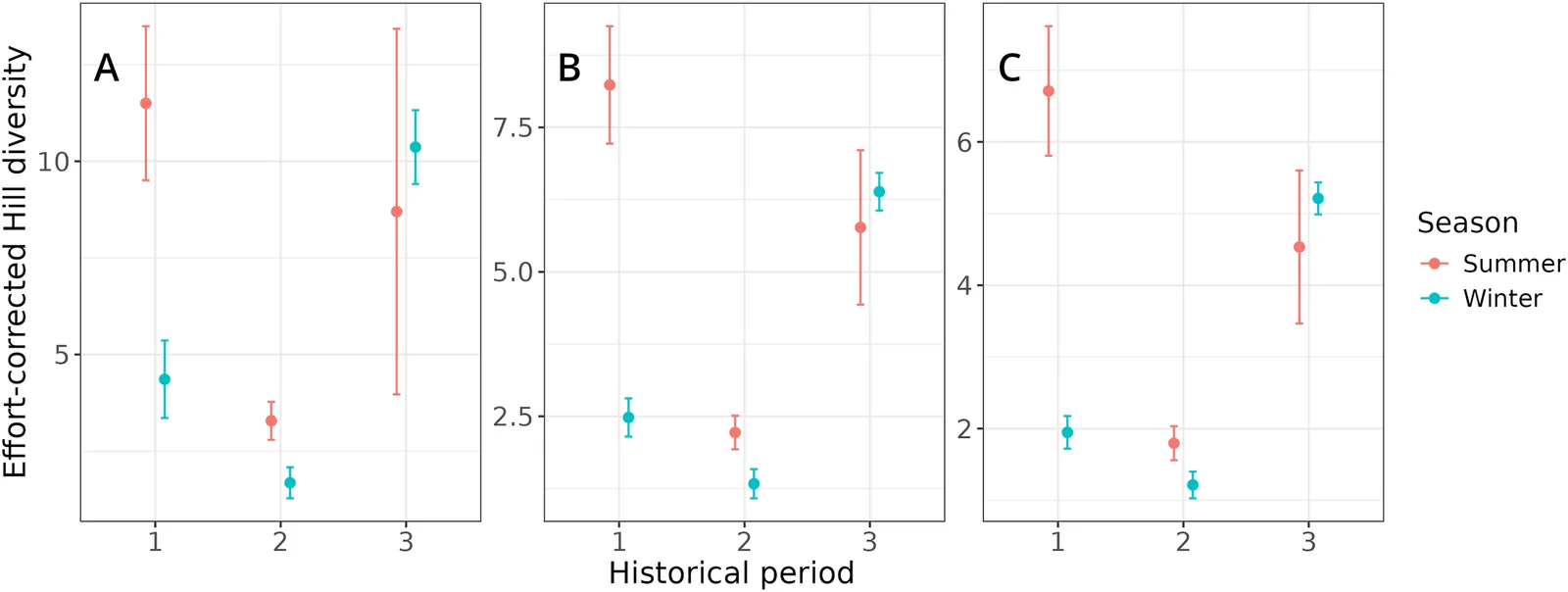
Effort-corrected diversity of helminth communities across historical periods and seasons. (A) Species richness (Hill number q = 0); (B) Exponential Shannon diversity (Hill number q = 1); (C) Inverse Simpson diversity (Hill number q = 2).

To evaluate how the structure of the helminth community varied across historical periods and seasons, helminth diversity was assessed using Hill numbers standardized by sample coverage (C = 0.946; Fig. 4). Here, the period-season combination was chosen to capture both long-term trends and potential seasonal differences. A plot for periods only is also included in the supplements (Supplementary Data, Fig. S1).

All three Hill diversity orders showed a consistent temporal pattern. The lowest diversity was observed in P2 and, in contrast, diversity values were substantially higher in P1 and P3.

Species richness (q = 0) was highest in P1 and P3 and markedly reduced in P2. Seasonal differences were evident in P1, where richness was considerably higher in summer than in winter. In P3 the pattern was reversed, with winter samples showing slightly higher richness than summer samples, although confidence intervals overlapped and, in that case, which can be also explained by uneven sampling (12 in summer versus 133 in winter).

Abundance-weighted diversity indices showed similar trends. Both the exponential Shannon diversity (q = 1) and the inverse Simpson index (q = 2) indicated reduced diversity in P2 compared with the other periods. In P1, summer community was considerably more diverse than the winter one, whereas in P3 diversity values were comparable between seasons and remained relatively high. The sample size- and coverage-based rarefaction and extrapolation curves for each period are presented in Supplementary Data, Fig. S2.

### 3.4 Changes in helminth community composition

**Fig. 5:**
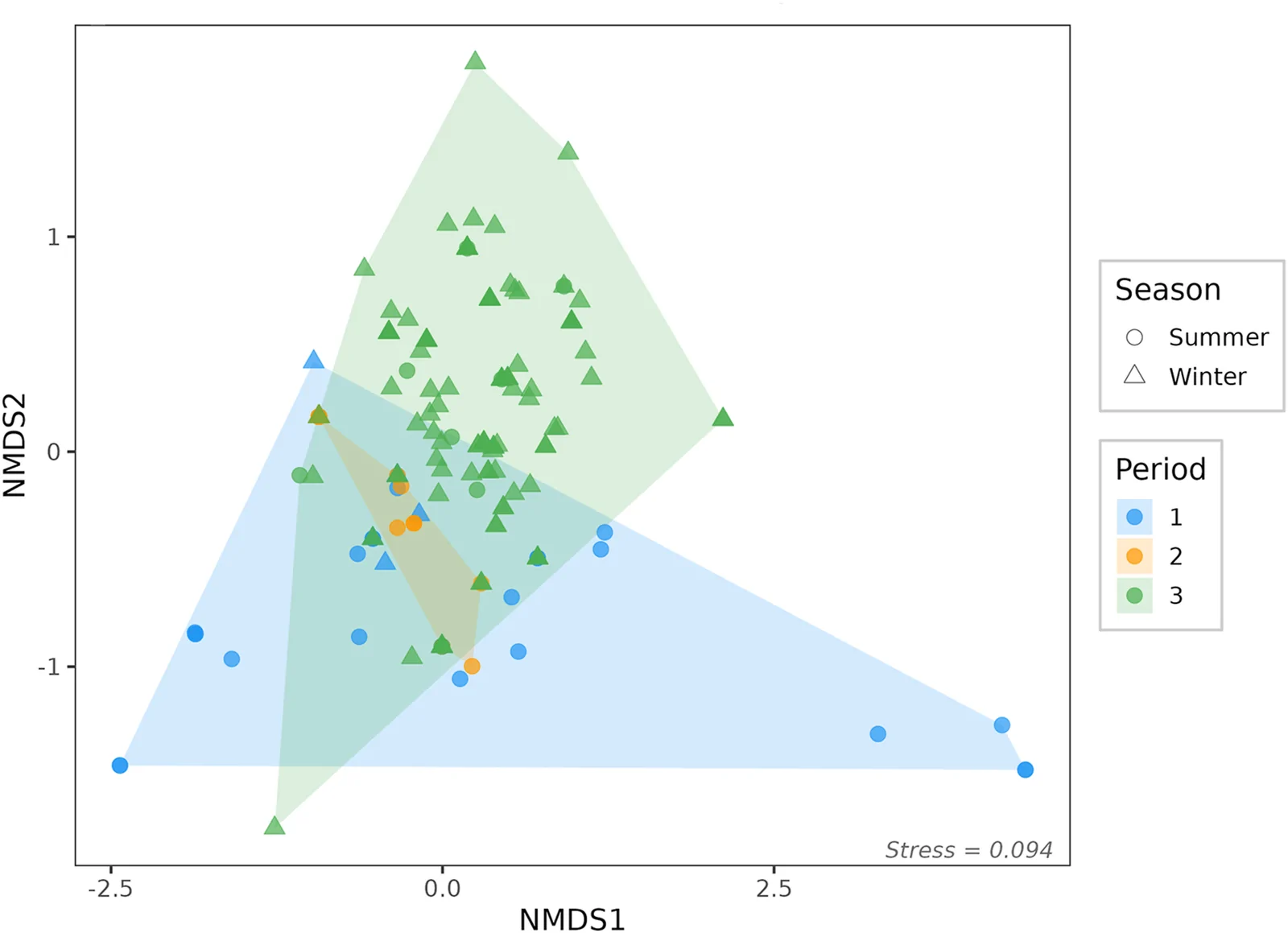
nMDS based on Sørensen dissimilarity matrix for helminth infracommunities.

The nMDS based on Sørensen dissimilarity visualization of helminth intracommunities showed some dissimilarity among historical periods (Fig. 5). There was a partial separation of samples, with P1 occupying a more distinct region of the ordination space. Samples from P2 and P3 overlapped substantially. P3 also showed a wider spread of points. A similar picture was observed using nMDS based on Bray-Curtis dissimilarity (Supplementary Data, Fig. S3).

ANOSIM indicated a significant but moderate separation of helminth communities among historical periods (R = 0.213, p = 0.0001). A similar but weaker pattern was observed for season (R = 0.119, p = 0.0001).

Consistent with this, PERMANOVA analyses based on Sørensen and Bray distances showed that helminth community composition varied significantly among historical periods. Additional repeated subsampling analyses with balanced sample sizes among historical periods produced highly similar results, which confirmed that the temporal signal was robust to unequal sampling effort. Across Bray subsampling iterations, historical period explained on average ∼11.0% of the variation (mean R^2^ = 0.110) and remained significant in 100% of runs (median p = 0.001), whereas season explained ∼2.5% of the variation and was significant only in 25.5% of runs. Results based on Sørensen distances were very similar. Historical period explained ∼17.9% (mean R^2^ = 0.179) of the variation and was significant in 100% of runs.

Pairwise PERMANOVA showed that P3 composition differed significantly from both P1 and P2 (p_adj = 0.00015), with stronger separation from P1 (R^2^ = 0.136) than from P2 (R^2^ = 0.110). In contrast, P1 and P2 did not differ significantly (p_adj = 0.073).

These temporal differences were robust to the spatial structure of the sampling. When sampling region was included as a covariate, historical period remained a significant predictor of community composition (Sørensen, R^2^ = 0.117, p < 0.001), of a magnitude comparable to region itself (R^2^ = 0.104, p < 0.001); the same held for Bray-Curtis distances. In addition, the two localities sampled across periods each showed significant compositional turnover on their own: at Kyiv, composition differed between P1 and P3 (R^2^ = 0.203, p < 0.001), and at Askania-Nova between P2 and P3 (R^2^ = 0.268, p < 0.001). We also tested for differences in multivariate dispersion among periods. Dispersion did not differ for abundance-based (Bray-Curtis) communities (F = 2.75, p = 0.067), which supports compositional differences rather than changes in within-group variability. In contrast, dispersion differed for presence/absence (Sørensen) communities (F = 5.01, p = 0.007), consistent with the wider compositional spread of P3. Sørensen differences could therefore be interpreted as a combination of a location shift and greater within-period heterogeneity in the most recent period.

SIMPER analysis showed that differences between historical periods were driven mainly by a few dominant taxa (Table 2). In all pairwise comparisons, the largest contributions came from *S. affinis, A. anthuris*, and *B. resectum*. Comparisons involving P3 also showed substantial contributions from *E. frugilegi* and *M. helix*.

**Table 2:** SIMPER results showing taxa contributions to differences in helminth communities between each pair of historical periods.

#	Species	Average	Percent	Cumulative
	**P1 vs P2**			
1	*Spiniglans affinis*	0.184	28.84	28.84
2	*Acuaria anthuris*	0.149	23.37	52.21
3	*Baruscapillaria resectum*	16.04	14.75	68.25
4	*Plagiorhynchus cylindraceus*	0.032	5.05	73.30
6	*Sphaerirostris picae*	0.0321	4.95	78.25
7	*Passerilepis crenata*	0.028	4.32	82.57
8	*Prosthogonimus ovatus*	0.026	4.05	86.62
	**P1 vs P3**			
1	*Eucoleus frugilegi*	0.157	18.30	18.30
2	Spiniglans affinis	0.145	16.85	35.16
3	*Acuaria anthuris*	0.121	14.10	49.26
4	*Microtetrameres helix*	0.112	13.00	62.26
5	*Baruscapillaria resectum*	0.098	11.39	73.65
6	*Plagiorhynchus cylindraceus*	0.029	3.43	77.08
7	*Eucoleus contortus*	0.023	2.69	79.77
8	*Prosthogonimus ovatus*	0.023	2.63	82.40
9	*Sphaerirostris picae*	0.021	2.46	84.85
10	*Eucoleus corvicola*	0.016	1.91	86.76
11	*Microtetrameres contorta*	0.015	1.70	88.46
	**P2 vs P3**			
1	*Spiniglans affinis*	0.186	22.82	22.82
2	*Eucoleus frugilegi*	0.154	18.85	41.68
3	*Microtetrameres helix*	0.110	13.41	55.09
4	*Baruscapillaria resectum*	0.106	12.99	68.08
5	*Acuaria anthuris*	0.106	12.99	81.06
6	*Passerilepis crenata*	0.025	3.05	84.12
7	*Eucoleus contortus*	0.023	2.78	86.89
8	*Dilepis undula*	0.020	2.49	89.39

Indicator species analysis (Table 3) identified *E. frugilegi* (IndVal = 0.77, p = 0.001) and *M. helix* (0.67, p = 0.001) as significant indicators of P3. *Passerilepis crenata* (Goeze, 1782) (0.31, p = 0.018) was associated with P2, and *Prosthogonimus ovatus* (Rudolphi, 1803) (0.28, p = 0.025) with P1. *S. affinis* (0.68, p = 0.001) was associated with P1 and P2, but not P3. This can indicate a shift in community composition toward dominance of *E. frugilegi* and *M. helix* species in the most recent period.

**Table 3:** Indicator species for each historical period, showing strength of association (IndVal) and significance (p).

Species	Period	IndVal	p
*Eucoleus frugilegi*	P3	0.765	0.001
*Spiniglans affinis*	P1, P2	0.679	0.001
*Microtetrameres helix*	P3	0.675	0.001
*Passerilepis crenata*	P2	0.313	0.018
*Prosthogonimus ovatus*	P1	0.284	0.025

### 3.5 The temporal dynamics of focal species

**Fig. 6:**
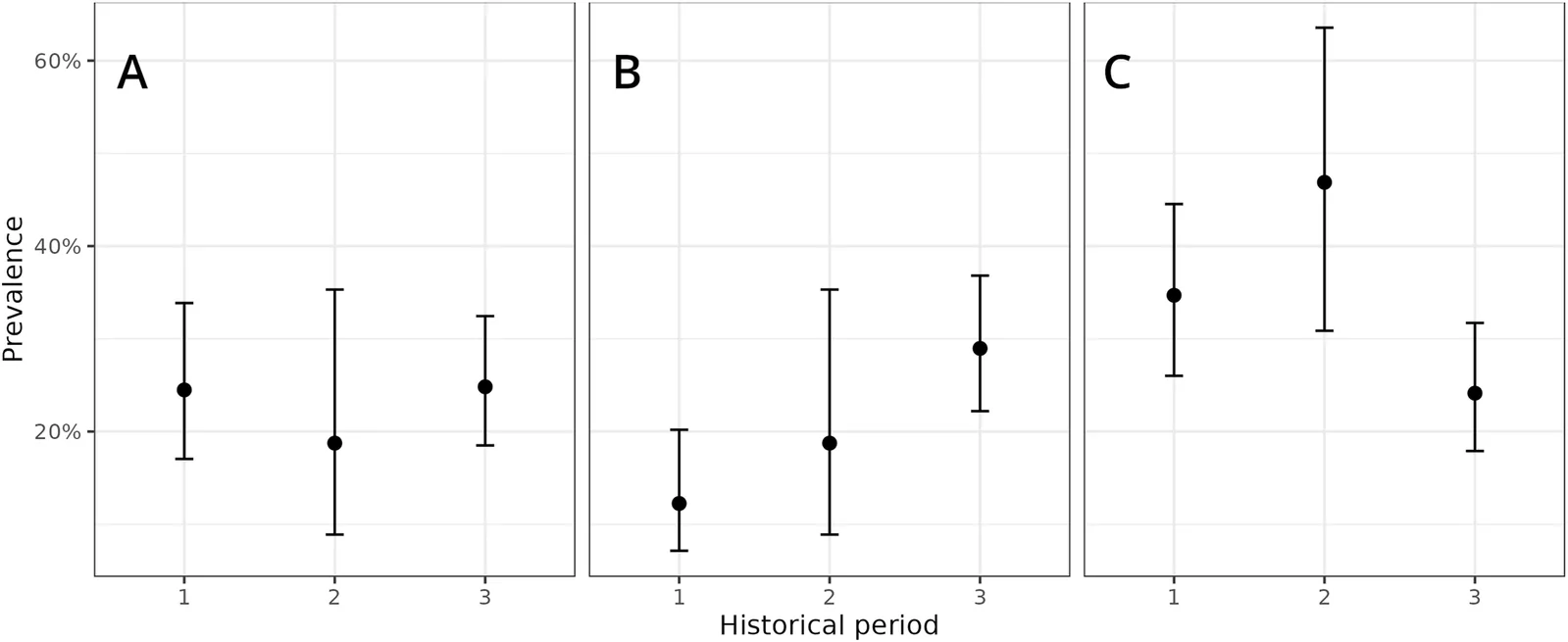
Prevalence of the three focal helminth species across historical periods (P1–P3), shown as proportions of infected hosts with Wilson 95% confidence intervals. A: *Acuaria anthuris*; B: *Baruscapillaria resectum*; C: *Spiniglans affinis*.

The prevalence of the three focal species for each historical period was calculated separately (Fig. 6).

Logistic regression showed no significant temporal or seasonal changes in *A. anthuris*. In contrast, prevalence increased significantly in *B. resectum* in P3 relative to P1 (OR = 2.87, p = 0.015), with no seasonal effect detected. For *S. affinis*, the additive model was preferred over the interaction model, indicating that temporal and seasonal effects acted independently. Infection probability was significantly higher in winter across periods (OR = 4.46, p < 0.001) and declined markedly in P3 relative to P1 (OR = 0.26, p < 0.001), whereas P2 did not differ significantly from P1 (OR = 2.28, p = 0.064). Thus *S. affinis* combined a consistent winter-associated increase with an overall decline in the most recent period, while its winter–summer difference remained similar across periods.

## 4 Discussion

### 4.1 Helminth fauna and taxonomic composition

The present study provides a comparative analysis of the helminth fauna of the rook across three historical periods spanning over seven decades (1950–2024), encompassing a total of 30 helminth species. The dominance of nematodes (13 species) over trematodes (10), cestodes (5), and acanthocephalans (2) is consistent with patterns observed in omnivorous passerines with terrestrial foraging habits, which are frequently exposed to soil-dwelling invertebrate intermediate hosts (Smith and Fedynich, 2012). The presence of *Cylicodontophorus bicoronatus* (Looss, 1900) – a nematode typically associated with ungulates – as an accidental parasite underscores the opportunistic nature of helminth transmission in a generalist corvid that forages across diverse habitats including agricultural fields and pastures.

### *4.2* *Diversity patterns across historical periods*

Coverage-standardised Hill number diversity estimates revealed a consistent pattern: diversity was markedly reduced in P2 relative to both P1 and P3, potentially reflecting a genuine depression of the helminth community during the mid-period (1975–1999). Critically, no trematode specimens were recorded during P2. Trematodes are generally regarded as among the most environmentally sensitive helminths (Shea et al., 2012; Stout et al., 2022; Taglioretti et al., 2018), and their complete absence during this period warrants explanation.

This temporal collapse of the helminth community coincides with the peak of agrochemical intensification in the USSR. Rook nesting colonies were closely associated with agricultural land throughout this period – in the 1980s, 75.6% of nesting colonies in central Ukraine were located within or very close (<1.5 km) to agricultural fields (Poluda et al., 2023). The USSR's agrochemical intensification programme reached full momentum in the 1970s–1980s, when major Western chemical corporations entered the Soviet market through trade and research agreements with the Ministry of Agriculture (Coudreau, 2024). Free-living larval stages of trematodes – miracidia and cercariae – exhibit significant mortality upon contact with certain pesticides even at sub-recommended concentrations, with sharp declines in the number of infective stages documented (Griggs and Belden, 2008; Gustafson et al., 2016; Puissant et al., 2021). Agricultural chemicals, particularly pesticides, can also affect free-living larval stages of nematodes, causing mortality even at relatively low concentrations (Sabo et al., 2009). Another possible cause of the collapse of trematode communities and the decline in the abundance and diversity of other helminth groups during P2 may have been the concurrent, insecticide-driven collapse of invertebrate diversity and abundance, including insects and soil-dwelling oligochaetes, which serve as intermediate hosts for many helminth taxa; this may have disrupted transmission cycles and exerted an indirect depressive effect on the helminth community as a whole (Ewald et al., 2015; Hallmann et al., 2017; Miglani and Bisht, 2019).

Long-term studies of helminth fauna dynamics in birds from Central Europe demonstrate that helminth communities in this host group may respond differently to environmental changes depending on the ecology of the bird species. In particular, substantial simplification of trematode assemblages, and even the disappearance of most previously common species, has been documented in waterbirds and species associated with agricultural landscapes (Sitko and Heneberg, 2020, 2021). For example, in black-headed gulls, approximately 67% of trematode species recorded in the 1960s were absent in the 2010s, which the authors attribute to disruptions of trophic networks and shifts in feeding behaviour, including increased reliance on anthropogenic food sources (Sitko and Heneberg, 2020). Only a few cosmopolitan and generalist helminth species persisted, and even in these, a decline in abundance of dominant species was observed (Sitko and Heneberg, 2021).

However, not all bird groups exhibit similar trends. In aerial insectivorous species, such as swallows, swifts, and flycatchers, long-term observations have not revealed a simplification of helminth communities. On the contrary, in some helminth species an increase in their intensity of infection and the appearance of new helminth species in later periods have been recorded, indicating a complex and context-dependent nature of changes in the helminth component community (Sitko and Heneberg, 2023).

The pattern observed in the rook thus represents a third, distinct type of helminth community response – one characterised by end-century depression followed by recovery and restructuring. Although this type of dynamics had not previously been recorded in birds, it has been observed in other host groups, notably mammals – for example, a non-linear decline-and-recovery pattern tied to host population collapse and subsequent recovery in striped dolphins *Stenella coeruleoalba* (García-Gallego et al., 2023), and compositional restructuring without directional diversity loss in bank voles *Myodes glareolus* (Grzybek et al., 2015), consistent with the patterns observed in rooks in Ukraine.

The increase in helminth diversity during P3 may be partly linked to the abandonment of Soviet agricultural practices following the political and economic transitions of the 1990s. However, the subsequent development of large agroholdings, landscape-level standardisation through monocultures, and renewed intensification of chemical inputs complicates this interpretation (Smaliychuk et al., 2016). Additional drivers of community change may include industrialisation, urban expansion, and the increasing availability of anthropogenic food resources – including solid waste landfills and other subsidised food sources – which may have altered the dietary composition of rooks and consequently the transmission pathways available to their helminth species.

The importance of these factors is likely amplified by the ecological characteristics of the rook itself. The species has a long-established association with agricultural landscapes and human settlements, documented since at least the 19th century. Historical sources indicate that in Ukraine rooks nested at forest edges, small groves, and villages, typically in close proximity to fields (Kessler, 1851; Khranevych, 1925). By the second half of the 20th century, virtually all colonies in central Ukraine were closely associated with human settlements and agricultural lands (Poluda et al., 2023). Unlike many other corvids that have urbanised only in recent decades, the rook has long nested and wintered in anthropogenically transformed environments. This prolonged exposure suggests that transformations of its helminth community may have begun well before systematic helminthological surveys were established, meaning that the contemporary helminth community of the rook likely represents a secondary, reorganised community shaped by decades of anthropogenic pressure rather than a primary natural complex.

### 4.3 Shifts in community composition

Ordination and multivariate analyses demonstrated significant differences in helminth community composition among historical periods, with historical period explaining approximately 17.9% of the total variation based on Sørensen dissimilarity. The tight clustering of P2 samples, overlapping with the cores of both other periods, may reflect the retention of widespread, generalist species typical of rooks and the loss of less common satellite species. Homogeneous multivariate dispersion among periods for abundance-based communities supports the interpretation of genuine temporal turnover rather than differences in within-period variability. The persistence of this pattern after accounting for sampling region, and its independent recovery within the two localities sampled across periods, further supports a temporal rather than purely spatial explanation for the observed changes.

P3 was strongly characterised by *E. frugilegi* (IndVal = 0.77) and *M. helix* (IndVal = 0.67) – two nematodes that, despite being considered common rook parasites, were entirely absent from the material collected in 1950–1999. Notably, *E. frugilegi* was originally described from rooks in 1962 (Czaplinski, 1962), yet was not recorded in any examined material during 1950–1999, and by the most recent 25-year period had become the dominant parasite, reaching a prevalence of 49.7%. *Microtetrameres helix*, similarly, absent in earlier periods, reached a prevalence of 38.6% in P3. Conversely, *S. affinis* – a cestode associated with P1 and P2 – was no longer an indicator taxon in P3, suggesting a relative decline in its importance within the community.

All helminth material from P1 and P2 held in the institutional collection was re-examined in full, and neither *E. frugilegi* nor *M. helix* was detected in any archived specimen, confirming that their absence from earlier periods is not a detection artifact. The emergence of these species as dominants in the rook helminth community is most likely attributable to one of two processes: a range expansion – particularly plausible for *E. frugilegi*, which has been recorded predominantly in Europe – or niche replacement, whereby incoming species occupied vacancies left by taxa that declined in earlier periods. The latter mechanism is supported by the case of *M. helix*, which appears to have replaced *Microtetrameres inermis* (Linstow, 1879) – a species recorded exclusively in P1 and sharing the same site of infection – suggesting a shift within the *Microtetrameres* component of the community rather than an entirely novel colonisation event. The emergence and dominance of the nematode *E. frugilegi* in rooks during P3 is also likely related to the biology and ecology of the helminth. This nematode has a high specificity for its intermediate host, the earthworm *Allolobophora chlorotica* (Savigny, 1826), as was established experimentally (Rietschel, 1973). The oligochaete *A. chlorotica* is a common species in agricultural soils, belongs to the endogenic ecological group of earthworms, and is characterised by resistance to ploughing and soil-applied pesticides (Curry, 2004; Diallo et al., 2023). These oligochaetes tolerate frost and soil stress from grazing cattle less well than other earthworms (Buslenko and Ivantsiv, 2020; Kunah et al., 2010), so the warm winters observed during P3 and the reduction in cattle numbers likely increased the survival of *A. chlorotica*. This likely enabled this earthworm species to compete with other species and, through its widespread presence in the soil, maintain the circulation of *E. frugilegi*.

Nevertheless, all patterns described here remain statistically supported observations, and the underlying mechanisms driving these shifts cannot be established with certainty from the available data alone.

### 4.4 Focal species dynamics

Four helminth species were recorded across all three periods – *A. anthuris, B. resectum, S. affinis*, and *S. picae* – of which only the first three had sufficient infection parameters for temporal analysis.

*Acuaria anthuris* showed no significant temporal or seasonal changes, consistent with its status as a widespread, non-specific corvid parasite. In contrast, *B. resectum* prevalence increased significantly in P3 relative to P1 despite no seasonal effect, possibly reflecting its monoxenous transmission (Okulewicz and Frońska, 1998), which may reduce its vulnerability to trophic-network disruptions affecting other taxa. The cestode *S. affinis,* considered a rook-specific helminth (Salamatin, 1999), showed the most pronounced changes: overall infection levels declined by P3, and its strong winter-associated infection probability in P1 weakened significantly, while summer prevalence remained relatively stable.

These results indicate that species persistence within the helminth community does not guarantee stability of its epidemiological profile, as infection dynamics can vary substantially with transmission mode and species-specific ecology.

### 4.5 Seasonal changes

Seasonal asymmetry in diversity is also informative. During P1, summer helminth communities were significantly richer and more diverse than winter ones, which is consistent with increased availability of invertebrate intermediate hosts in the warmer months and a more varied diet during the breeding season (Chernobaj, 1969b; Zekhnov, 1949). The apparent shift in trends during P3 is most likely an artifact of highly uneven seasonal sampling (12 summer vs. 133 winter individuals) and should not be overinterpreted. Although coverage-based standardization partially corrects for sampling effort, residual variation in sample sizes between periods and seasons may still affect the accuracy of diversity estimates.

At the same time, these results may partly reflect real ecological changes in recent decades. In particular, the availability of helminth intermediate stages in winter may have increased due to the reduction in the duration and stability of snow cover. Climate change has led to a shortening of the snow cover period and an increase in the number of snow-free days in Europe, including Ukraine (Amihăesei et al., 2024; Pyasetska and Shcheglov, 2024; Szyga-Pluta, 2022; Young, 2023). This supports year-round foraging activity of birds on open ground and, accordingly, may allow the persistence of transmission cycles of geohelminths and some helminths with complex life cycles.

## CRediT authorship contribution statement

**Valeriia Dupak:** Writing – original draft, Methodology, Funding acquisition, Data curation, Conceptualization. **Valeriia Telizhenko:** Writing – review & editing, Visualization, Methodology, Formal analysis. **Oksana Greben:** Writing – review & editing, Resources, Methodology, Data curation.

## Funding

This study was financially supported by the 10.13039/100018227National Research Foundation of Ukraine (project number 2023.03/0068).

## Declaration of competing interest

The authors declare that they have no known competing financial interests or personal relationships that could have appeared to influence the work reported in this paper.
