# Supplementary material for: Seventy-five years of change: Long-term reorganisation of helminth communities in the rook (Corvus frugilegus) in Ukraine
Source: Int J Parasitol Parasites Wildl. 2026 Sep 16;31:101289. doi: 10.1016/j.ijppaw.2026.101289 (PMC13602256; doi:10.1016/j.ijppaw.2026.101289)
Supplement: Supplementary file 1 [file mmc1.docx]

**Supplementary material for**

**Seventy-five Years of Change: Long-term Reorganisation of Helminth Communities in the Rook in Ukraine under Anthropogenic Pressure**

Valeriia Dupak, Valeriia Telizhenko, Oksana Greben

**Figure S1.** Effort-corrected diversity of helminth assemblages across historical periods. (A) Species richness (Hill number q = 0); (B) Exponential Shannon diversity (Hill number q = 1); (C) Inverse Simpson diversity (Hill number q = 2)

**Figure S2.** Rarefaction and extrapolation curves of Hill diversity for historical assemblages. (A) Sample-size-based standardization and (B) coverage-based standardization for Hill numbers q = 0 (richness), q = 1 (exponential Shannon diversity), and q = 2 (inverse Simpson diversity). Solid lines represent rarefaction, dashed lines represent extrapolation, and shaded ribbons indicate 95% confidence intervals.

**Figure S3**. nMDS based on Bray-Curtis dissimilarity matrix for parasite assemblages
